# National Teen Driver Safety Week — October 19–25, 2014

**Published:** 2014-10-17

During 2003–2012, the number of teens aged 13–19 years who died in motor vehicle crashes declined by 50%, from 5,718 to 2,823 (1). During the same period, the rate of passenger vehicle drivers aged 16–19 years involved in fatal crashes decreased by 52%, from 35.1 to 16.8 per 100,000 persons (1). Despite these encouraging trends, motor vehicle crashes remain the leading cause of death for teens. Among teens who died in passenger vehicle crashes in 2012, approximately 60% were not wearing a seat belt (1). Parents can be good role models by always wearing their seat belts and insisting that their teen drivers and all of their passengers always buckle up.

Graduated driver licensing (GDL) programs are widely credited with contributing to declines in teen crash deaths. Evaluations of GDL programs have demonstrated a 20%–40% reduction in crash risk among the youngest drivers (2,3). GDL programs provide longer practice periods, limit driving under high-risk conditions for newly licensed drivers, and require greater participation of parents in their teen’s learning-to-drive process.

This year, during National Teen Driver Safety Week, CDC is releasing an updated Parents Are the Key campaign website, available at http://www.cdc.gov/parentsarethekey. Using the science behind GDL, Parents Are the Key provides families with tools and tips to help keep their teen drivers safe, including a parent-teen driving agreement. Additional information regarding National Teen Driver Safety Week is available from the National Highway Traffic Safety Administration at http://www.trafficsafetymarketing.gov/teens.
